# HDAC2 interacts with microRNA-503-5p to regulate SGK1 in osteoarthritis

**DOI:** 10.1186/s13075-020-02373-y

**Published:** 2021-03-09

**Authors:** Zheng Wang, Nan Zhou, Wengang Wang, Yangke Yu, Lei Xia, Ning Li

**Affiliations:** Department of Orthopaedics, The First Affiliated Hospital of Zhengzhou University, No. 1, Eastern Jianshe Road, Zhengzhou, 450000 Henan Province People’s Republic of China

**Keywords:** Osteoarthritis, Histone deacetylase 2, MicroRNA-503-5p, Serum- and glucocorticoid-inducible kinase-1, Chondrocyte

## Abstract

**Background:**

Osteoarthritis (OA) is a disabling joint disease that causes articular cartilage degeneration. It has been implicated that altered expression of histone deacetylase 2 (HDAC2) is found in patients with OA. However, the specific role of HDAC2 in the development of OA still remains enigmatic. Hence, we sought to characterize the functional relevance of HDAC2 in the development of OA.

**Methods:**

Anterior cruciate ligament surgery was performed to generate the rat model of OA. Luciferase assay was performed to evaluate the relationship between microRNA-503-5p (miR-503-5p) and serum- and glucocorticoid-inducible kinase-1 (SGK1). Functional experiments were conducted to examine the functional significance of miR-503-5p, histone deacetylase 2 (HDAC2), and SGK1 on the progression of OA by determining proliferation, apoptosis, and expression of apoptosis-associated proteins and inflammatory cytokines.

**Results:**

HDAC2 could inhibit miR-503-5p expression. SGK1 was the target gene of miR-503-5p. Upregulation of miR-503-5p or silencing of HDAC2 contributed to enhanced proliferation, suppressed apoptosis (reduced expression of Caspase-3 and Bax but elevated expression of Bcl2), and promoted inflammation in chondrocytes of OA rats.

**Conclusion:**

In conclusion, our study demonstrated that HDAC2 could promote OA through miR-503-5p/SGK1 axis, which might function as a therapeutic target for OA treatment.

## Background

Osteoarthritis (OA) is one of the most common rheumatism characterized by cartilage rupture and synovial inflammation [1]. Its prevalence and incidence are expected to rise as life expectancy increases, bringing a heavy burden on society [2]. The development of OA is attributed to major risk factors including gender, age, obesity, and huge mechanical stress [3]. Due to limited knowledge with regard to the exact molecular mechanism involved in the degradation of cartilage matrix and the development of OA, there is no effective way to treat OA, apart from total joint replacement [4]. Recent advances in epigenetic research are beneficial to better understanding the pathogenesis of OA [5]. Luckily, microRNAs (miRNAs) have been demonstrated to be a key regulator in the development of OA and may serve as novel therapeutic target for OA treatment [6, 7].

In recent years, many abnormally expressed miRNAs have been reported to participate in the occurrence and development of OA [8]. It has been reported that there are 17 miRNAs lowly expressed in the anterior cruciate ligament of OA [9], especially miR-503-5p compared with that of normal people [10]. Therefore, we attempted to examine the specific effect of miR-503-5p in the progression of OA. Moreover, miR-503-5p capable of targeting serum- and glucocorticoid-inducible kinase-1 (SGK1) was verified by luciferase assay in the present study. SGK1 belongs to a serine/threonine kinase that acts under acute transcriptional control of multiple stimuli, including serum and glucocorticoids, which is able to regulate inflammation and cell proliferation as well as apoptosis [11]. More importantly, findings obtained from a study showed that SGK1 is highly expressed in chondrocytes of OA and inhibition of SGK1 can reduce IL-1β-induced chondrocyte anabolic and catabolic imbalance in human chondrocytes [12]. Additionally, miR-576-3p is able to suppress cell migration and invasion via targeting SGK1 in lung cancer [13]. Here, we inferred that miR-503-5p could regulate the progression of OA by targeting SGK1. Furthermore, downregulation of miR-503-5p has been achieved by histone deacetylases (HDACs) via binding to the promoter of miR-503-5p and inhibiting H3K27ac expression [14]. HDAC2 is one of member of HDACs family, which are a class of enzymes that remove acetyl groups from histones and other nuclear proteins, thus leading to chromatin condensation as well as transcriptional repression [15]. Intriguingly, previously conducted study suggested that HDAC2 participated in regulating phenotypic progression of chondrocyte in OA [16]. Based on previous studies, we hypothesized that HDAC2 could potentially regulate the miR-503-5p/SGK1 axis, by which affected the development of OA.

## Materials and methods

### Ethics statement

The study was conducted under the approval of the Ethics Committee of the First Affiliated Hospital of Zhengzhou University. All participants signed informed consent. The animal experiments were performed in compliance with the Guide for the Care and Use of Laboratory Animals of the National Institutes of Health.

### Sample recruitment

OA samples were surgically removed from 17 patients (8 males and 9 females aged 34–78 years with a mean age of 64.76 ± 3.05 years, Kellgren-Lawrence grade III–IV) during knee replacement surgery at the First Affiliated Hospital of Zhengzhou University. Samples derived from 17 healthy people (6 males and 11 females aged 39–63 years with a mean age of 48.24 ± 9.06 years old) with amputation or corrective surgery were used as controls.

### Establishment of rat models of OA

Thirty-six-week-old Sprague-Dawley (SD) rats weighing 250–300 g were intraperitoneally injected with xylazine (7 mg/kg, Rompun; Bayer, Istanbul, Turkey) and ketamine hydrochloride (60 mg/kg, Ketalar; Parke-Davis, Istanbul, Turkey). Rats’ right knees were disinfected with polyvinyl iodide (Betadine, Eczacibasi, Turkey), and the pat bone skin incision was made on the inside of the joint. To expand the surgical field of view, an incision was made in the bone and the lateral thigh muscles. The anterior cruciate ligament was cut using a # 11 surgical blade followed by a positive anterior drawer test to ensure complete transection of the ligament. The retina was repaired and the skin was sutured separately. All surgical steps were conducted using magnification. Every 10 modeled rats were subcutaneously inoculated with miR-503-5p mimic or mimic NC (Hanbio Biotechnology Co., Ltd., Shanghai, China) via joint cavities of rats. For postoperative analgesia, fentanyl citrate (0.02 mg/kg, fentanyl; Abbott Laboratories, Abbott Park, IL, USA) was subcutaneously injected twice per day for 3 days after surgery.

### Culture and transfection of primary chondrocytes

Epiphyseal cartilage deriving from femurs and tibias was collected from 4-day-old male Sprague-Dawley rats followed by removing the perichondrium. After being washed twice in chilled phosphate-buffered saline (PBS) supplemented with 1% penicillin/streptomycin and fungizone, the epiphyseal cartilage was cultured in 15 mL PBS containing 0.1% ethylenediaminetetraacetic acid (EDTA) followed by a culture in 14 mL PBS containing 0.125% trypsin (Thermo Fisher Scientific, Waltham, MA, USA). After being resuspended and cultured in 0.3% collagenase (20 mL, Sigma-Aldrich, St. Louis, MO, USA), the supernatant was collected and then centrifuged followed by resuspension of chondrocyte pellet in chondrogenic medium. All cartilage was digested, and chondrocytes were filtered through a cell strainer (70 μm). After being washed, cells were seeded into 6-well plates at 1 × 10^7^ cells per well in Dulbecco’s modified Eagle’s medium (DMEM) containing 10% fetal bovine serum (FBS) and 50 μg/mL l-ascorbic acid. After 24 h, cells at about 50% confluency were washed twice with PBS and the medium was replaced for Opti-MEM (Thermo Fisher Scientific). Cells were transfected with miR-503-5p mimic, miR-503-5p inhibitor, oe-HDAC2, sh-HDAC2, mimic NC, and inhibitor NC, as per the instructions of Lipofectamine 2000 (Invitrogen, Carlsbad, CA, USA).

### Reverse transcription quantitative polymerase chain reaction (RT-qPCR)

Total RNA was extracted using TRIzol reagent (Beijing Solarbio Science and Technology Co., Ltd., Beijing, China). The primers used were synthesized by Takara (Dalian, China) (Table 1). RT was performed according to the instructions of cDNA (K1622, Beijing Yaanda Biotechnology Co., Ltd.) and miRNA RT kits (D1801, HaiGene, Harbin, China). Fluorescence-based qPCR assay was developed using PCR instrument (ViiA 7, Daan Gene Co., Ltd., of Sun Yat-sen University, Guangzhou, China). The transcription level of target genes was quantified by 2^−ΔΔCt^ method normalized to U6 and GAPDH.

**Table 1:** Primer sequences for RT-qPCR

Gene	Primer sequence
miR-503-5p	F: 5-TAGCAGCGGGAACAGTTCTGCAG-3
	R: 5-AACGCTTCACGAATTTGCGT-3
U6	F: 5-CTCGCTTCGGCAGCACA-3
	R: 5-AACGCTTCACGAATTTGCGT-3
GAPDH	F: 5-GGCATCCTGGGCTACACTGA-3
	R: 5-GAGTGGGTGTCGCTGTTGAA-3

*RT-qPCR* reverse transcription quantitative polymerase chain reaction, *miR-503-5p* microRNA-503-5p, *GAPDH* glyceraldehyde-3-phosphate dehydrogenase, *F* forward, *R* reverse

### Collection of OA rat samples

On the 20th day after surgery, 10 rats were euthanized by an anesthetic overdose. The articular cartilage tissue of the medial tibial plateau was stored at − 80 °C. The collected bone joints were diluted with 2 mL of 0.9% NaCl and filtered with a 1.2-μm filter, following adding 10% (v/v) protease and phospholipase inhibitor. The mixture was centrifuged to collect the supernatant, which was frozen at − 80 °C for cytokine analysis.

### Hematoxylin and eosin (HE) staining

Cartilage tissues of rats were fixed in 4% paraformaldehyde phosphate buffer for 12 h followed by conventional xylene dewaxing and hydration by gradient alcohol (anhydrous ethanol, 95% ethanol, 75% ethanol for 3 min each). Rat joint tissue pieces were boiled in 0.01 M citrate buffer for 15–20 min, sealed by goat serum blocking solution, and incubated to remove excess liquid. Tissues were stained with hematoxylin and differentiated by hydrochloric acid alcohol. After conventional dehydration and clear, tissues were sealed and observed under an inverted microscope.

### Lentivirus-mediated shRNA

OA cells expressing sh-SGK1 was conducted by transfecting lentiviral vector-based short interfering RNA (siRNA) plasmid (pILenti-siRNA-GFP, Applied Biological Materials, Richmond, BC, Canada) expressing short hairpin RNA (shRNA) duplexes targeting SGK1. Cells transfected with the empty vector served as a negative control (NC).

### Dual luciferase reporter gene assay

The wild type (WT) and mutant type (MUT) sequence of SGK1 3′-untranslated region (3′UTR) was artificially synthesized by GenePharma (Shanghai, China), namely PmirGLO-SGK1 3′UTR-WT and PmirGLO-SGK1 3′UTR-MU1, and subcloned into PGL3 promoter vector (Promega Corporation, Madison, WI, USA) containing luciferase reporter gene. Chondrocytes of OA rats and normal articular chondrocytes were seeded into 24-well plates. When the cell confluence reached about 70–80%, PmirGLO-SGK1 3′UTR-WT or PmirGLO-SGK1 3′UTR-MU1 (200 ng) were co-transfected with miR-503-5p mimic (200 ng) and Lipofectamine 2000 (Invitrogen, USA) into cells following the manufacturer’s instructions. The PmirGLO promoter vector (200 ng) was used as a control. After 48 h, the dual luciferase reporter system (Promega Corporation, USA) was used to measure luciferase activity.

### Western blot analysis

Cartilage tissue was ground and cell samples were washed with precooled PBS and were lysed in a cold radio-immunoprecipitation assay (RIPA) lysis buffer (Beyotime, Beijing, China). Protein concentration was detected using bicinchoninic acid (BCA) protein assay kit (Applygen, Beijing, China). All samples were treated with chondroitin enzymes ABC, keratinase, and keratinase II (Sigma-Aldrich) without the protease. Next, proteins were separated by 10% sodium dodecyl sulfate-polyacrylamide gel electrophoresis, transferred to a membrane of polyvinylidene fluoride (Thermo Fisher Scientific), and then blocked at room temperature. The membrane was probed with diluted primary antibodies to GAPDH (1:1000, ab8245, Abcam), SGK1 (ab43606), HDAC2 (Y461, ab32117), Bcl-2 associated X protein (Bax; E63, ab32503), B cell lymphoma-2 (Bcl-2; E18, ab32370), and Cleaved Caspase-3 (ab49822) overnight at 4 °C. The 1-h culture of cells was conducted after supplementing with secondary antibody labeled by horseradish peroxidase (HRP). Bands of protein were visualized using the enhanced chemiluminescence (BB-3501, Amersham-Pharmacia Biotech, Freiburg, Germany). Optical density of image was determined using ImageJ Software, with GAPDH as the internal reference.

### Enzyme-linked immunosorbent assay (ELISA)

According to the manufacturer’s instructions, the levels of IL-1β, IL-6, and TNF-α, which were considered as vital participators in OA development, were measured using an ELISA kit (R&D Systems, Minneapolis, MN, USA).

### Methyl-thiazoldiphenyl-tetrazolium (MTT) colorimetric assay

According to the instructions of MTT kit (Sigma-Aldrich), cells were cultured in 96-well plates at 2 × 10^5^, followed by addition of 0.5 mg/mL and 4 h incubation. The crystal was dissolved in 100 μL of dimethyl sulfoxide (DMSO). The optical density (OD) value of each well was measured at 570 nm on a Bio-Rad microplate reader (CA, USA).

### Flow cytometry

Flow cytometry was done using Annexin V-fluorescein isothiocyanate (AnV-FITC) kit (Sigma-Aldrich). Having been washed twice in PBS, cells were resuspended in a binding buffer. The cells were then incubated with AnV-FITC in darkness for 10 min. Ropiperidine iodide was used to adjust the final concentration to 1 mg/L. The stained cells were counted using FACScalibur (Becton Dickinson, Mountain View, CA, USA).

### Statistical analysis

With SPSS 21.0 statistical software (IBM Corp. Armonk, NY, USA), measurement data were expressed as mean ± standard deviation. Data between two groups were compared using *t* test while data among multiple groups by one-way analysis of variance (ANOVA) with Tukey’s post hoc test. A value of *p* < 0.05 was indicative of significant difference.

## Results

### miR-503-5p is poorly expressed in OA cartilage tissues

**Fig. 1:**
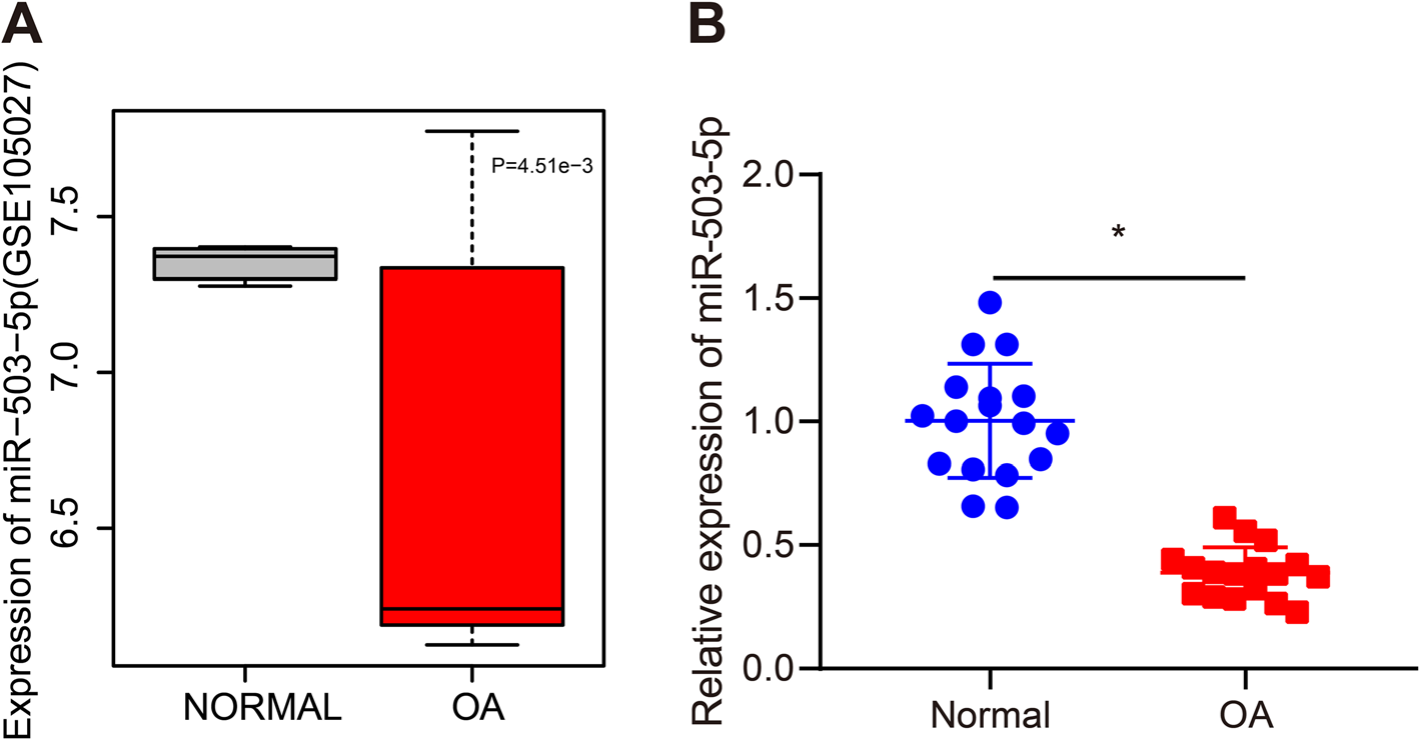
Low expression of miR-503-5p is found in OA. **a** Expression of miR-503-5p in OA samples and normal samples in OA-related microarray dataset GSE105027. **b** Expression of miR-503-5p in the cartilage tissues of patients with OA and normal people determined by RT-qPCR assay. *p < 0.05 vs. tissues of normal people. Measurement data were expressed as mean ± standard deviation. Data were compared using *t* test. The experiment was repeated three times

Previous bioinformatic analysis indicated low expression of miR-503-5p in OA cartilage tissues (Fig. 1a). Our results from RT-qPCR assay displayed that miR-503-5p was downregulated in the cartilage tissues of patients with OA than in those of normal people (Fig. 1b). The above results proved that miR-503-5p was lowly expressed in OA.

### miR-503-5p promotes the proliferation of rat primary chondrocytes

**Fig. 2:**
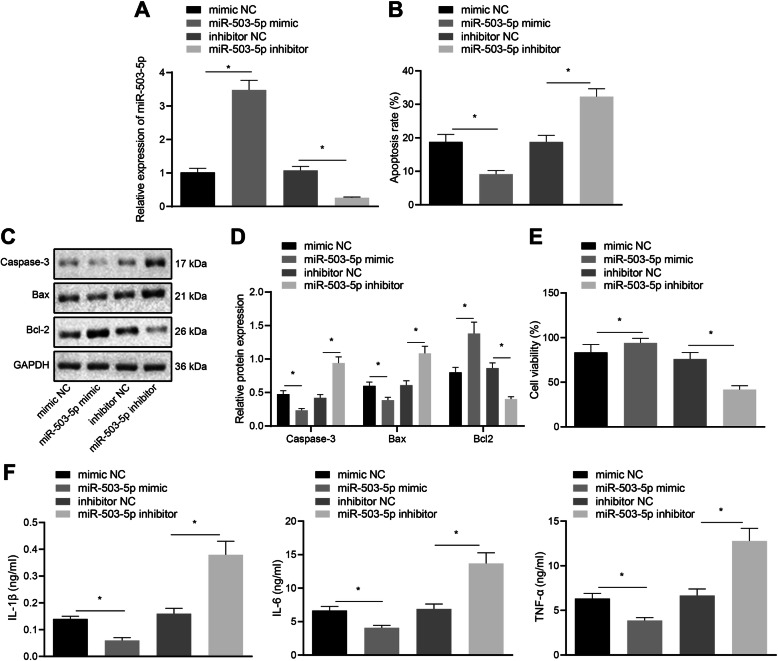
Overexpression of miR-503-5p results in promoted proliferation and reduced apoptosis of rat primary chondrocytes. **a** The expression efficiency of miR-503-5p verified by RT-qPCR assay. **b** Apoptosis of rat primary chondrocytes after overexpression or downregulation of miR-503-5p detected by flow cytometry. **c** The expression of Caspase-3, Bax, and Bcl2 in primary chondrocytes after overexpression or downregulation of miR-503-5p detected by Western blot analysis. **d** Statistics of **c**. **e** Proliferation of rat primary chondrocytes after overexpression or downregulation of miR-503-5p detected by MTT assay. **f** The expression of inflammatory cytokines in rat primary chondrocytes examined by ELISA. *p < 0.05. Measurement data were expressed as mean ± standard deviation. Comparisons among multiple groups were conducted by one-way ANOVA with Tukey’s post hoc test. The experiment was repeated three times

To further explore the effect of miR-503-5p on rat primary chondrocytes, RT-qPCR assay (Fig. 2a) results displayed that treatment of miR-503-5p mimic increased the expression of miR-503-5p in rat primary chondrocytes, while treatment of miR-503-5p inhibitor reduced the expression of miR-503-5p in rat primary chondrocytes. Flow cytometry results revealed that overexpression of miR-503-5p inhibited the apoptosis of primary chondrocytes, accompanied with reduced expression of Caspase-3 and Bax, and enhanced expression of Bcl2 (Fig. 2b–d). MTT results confirmed that overexpression of miR-503-5p promoted the proliferation of primary chondrocytes (Fig. 2e). Besides, ELISA suggested that upregulation of miR-503-5p inhibited the release of IL-1β, IL-6, and TNF-α (Fig. 2f). The above results demonstrated that miR-503-5p facilitated the proliferation of primary chondrocytes, inhibited the apoptosis of chondrocytes, and suppressed the secretion of inflammatory cytokines.

### Overexpression of miR-503-5p promotes proliferation and inhibits apoptosis of OA chondrocytes

**Fig. 3:**
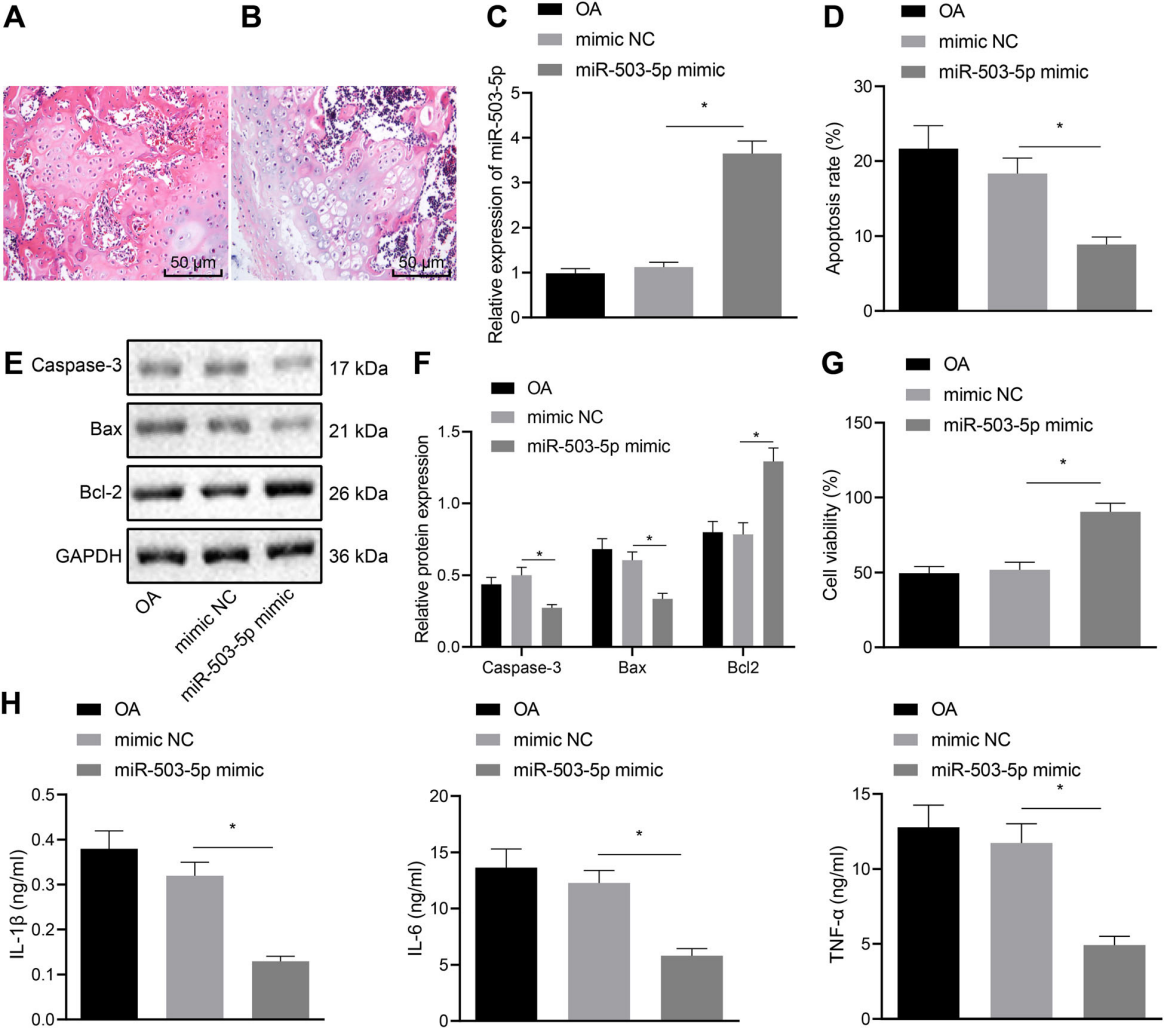
Upregulation of miR-503-5p facilitates proliferation and suppresses apoptosis of OA chondrocytes and promotes secretion of inflammatory cytokines. **a** Successful modeling of OA rats confirmed by Masson staining (50 μm). **b** Representative images of cartilage tissues of OA rats stained by Masson staining (50 μm). **c** Expression of miR-503-5p in OA rats measured by RT-qPCR assay. **d** Apoptosis in OA chondrocytes transfected with overexpressed miR-503-5p examined by flow cytometry. **e** Gray value analysis of apoptosis-related factors (Caspase-3, Bax, and Bcl2) in OA chondrocytes transfected with overexpressed miR-503-5p. **f** The protein expression of apoptosis-related factors (Caspase-3, Bax, and Bcl2) in OA chondrocytes transfected with overexpressed miR-503-5p measured by Western blot analysis (*n* = 10). **g** Proliferation of primary chondrocytes in OA rats treated with upregulated miR-503-5p detected by MTT assay (*n* = 10). **h** The expression of inflammatory cytokines in primary chondrocytes from OA rats treated with upregulated miR-503-5p assessed by ELISA. *p < 0.05. Measurement data were expressed as mean ± standard deviation. Data in compliance with normal distribution and homogeneity of variance between two groups were compared using *t* test. Comparisons among multiple groups were conducted by one-way ANOVA with Tukey’s post hoc test. The experiment was repeated three times

Next, the therapeutic effect of miR-503-5p on OA was explored. A positive anterior drawer test was performed to examine a rat model of OA. The results of Masson staining (Fig. 3a, b) showed that the cartilage tissue of rats was completely calcified. RT-qPCR revealed that the expression of miR-503-5p in cartilage tissues of OA rats was higher after overexpression of miR-503-5p (Fig. 3c). Besides, we also found that overexpression of miR-503-5p inhibited the apoptosis of chondrocytes in OA rats, accompanied with downregulated of apoptotic factors Caspase-3 and Bax but increased Bcl-2 (Fig. 3d–f). MTT assay showed that overexpressed miR-503-5p promoted chondrocyte proliferation in OA rats (Fig. 3g). Moreover, overexpressed miR-503-5p decreased the release of inflammatory factors IL-1β, IL-6, and TNF-α in cartilage tissue of OA rats (Fig. 3h). The above results demonstrated that overexpression of miR-503-5p was contributed to ameliorated OA.

### Overexpression of HDAC inhibits proliferation and promotes apoptosis of chondrocytes by downregulating miR-503-5p expression

**Fig. 4:**
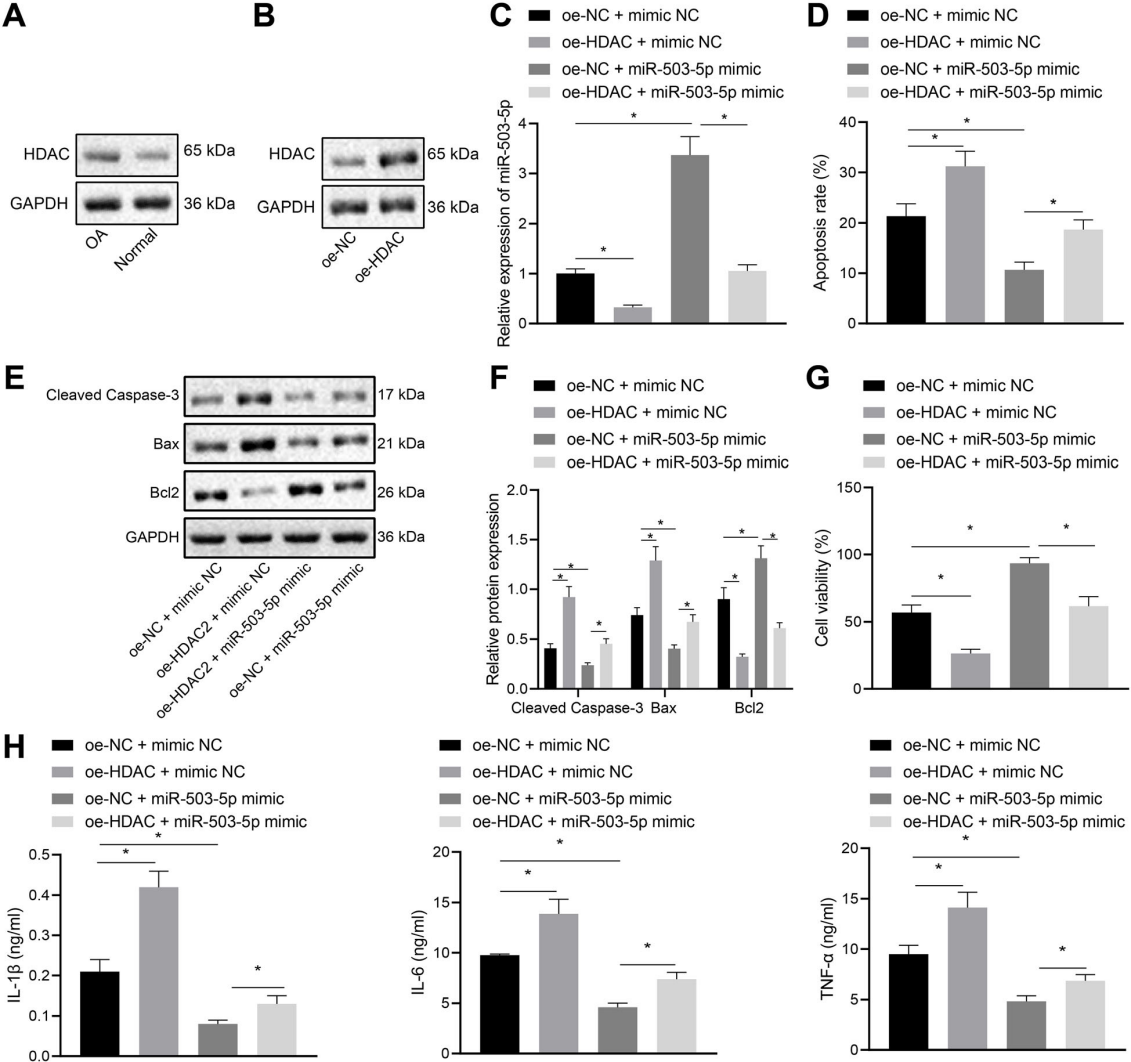
Overexpression of HDAC suppresses proliferation and stimulates apoptosis of chondrocytes by downregulating the expression of miR-503-5p. **a** Western blot analysis for determination of protein expression of HDAC2 in primary chondrocytes and OA chondrocytes. **b** Expression of HDAC in rat OA chondrocytes after overexpressing HDAC detected by Western blot analysis. **c** Expression of miR-503-5p in primary chondrocytes and OA chondrocytes examined by RT-qPCR assay. **d** Apoptosis in OA chondrocytes examined by flow cytometry. **e** Gray value analysis of apoptosis-related factors (Caspase-3, Bax, and Bcl2) in OA chondrocytes. **f** The protein expression of apoptosis-related factors (Caspase-3, Bax, and Bcl2) in OA chondrocytes detected by Western blot analysis. **g** Proliferation of primary chondrocytes in OA rats measured by MTT assay. **h** The expression of inflammatory cytokines in primary chondrocytes from OA rats assessed by ELISA. *p < 0.05, ^#^*p* < 0.05. Measurement data were expressed as mean ± standard deviation. Comparisons among multiple groups were conducted by one-way ANOVA with Tukey’s post hoc test. The experiment was repeated three times

It has been documented that HDAC2 binds to the promoter of miR-503-5p, inhibits H3K27ac, and decreases the expression of miR-503-5p [14]. Firstly, highly expressed HDAC2 was found in chondrocytes of OA (Fig. 4a). Treatment of oe-HDAC2 increased HDAC2 expression in chondrocytes (Fig. 4b). To validate the inhibitory effect of HDAC on miR-503-5p, we observed chondrocyte changes by overexpressing HDAC or miR-503-5p. Additionally, overexpression of HDAC inhibited miR-503-5p expression (Fig. 4c). Flow cytometry results showed that overexpression of HDAC promoted apoptosis of chondrocytes, while overexpression of miR-503-5p and HDAC together inhibited apoptosis (Fig. 4d). It was further confirmed by Western blot (Fig. 4e, f) and MTT assay (Fig. 4g) that overexpression of HDAC repressed the proliferation of chondrocytes and led to increased expression of apoptotic factors Caspase-3 and Bax as well as decreased Bcl-2 expression. In addition, overexpression of HDAC produced a promoting effect on the release of inflammatory factors (Fig. 4h). The above results demonstrated that overexpression of HDAC inhibited proliferation of chondrocytes, and promoted the release of inflammatory factors by inhibiting the expression of miR-503-5p.

### miR-503-5p targets SGK1

**Fig. 5:**
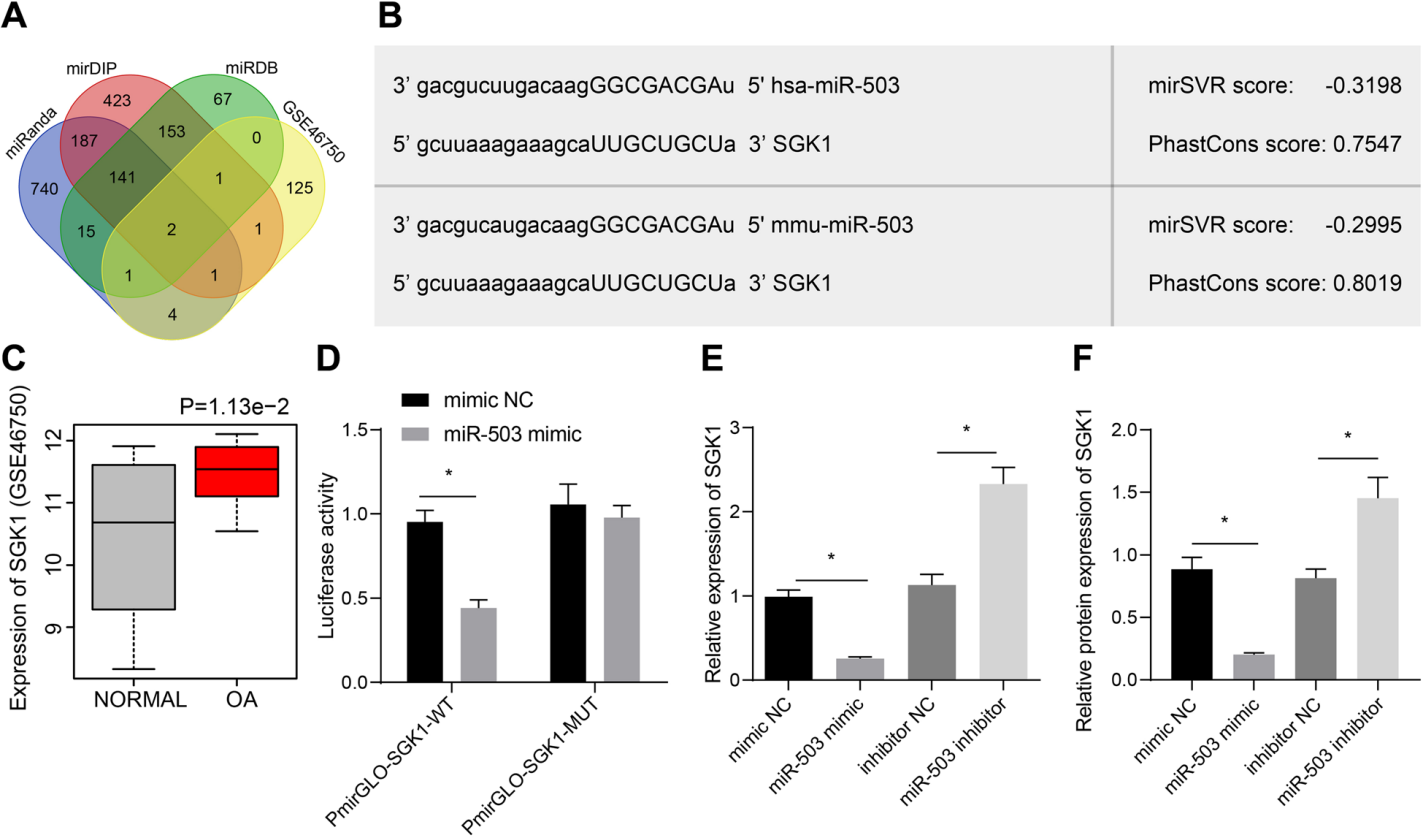
miR-503-5p downregulates SGK1 expression in OA. **a** Prediction of potential target genes of miR-503-5p by the miRanda database (http://www.microrna.org/microrna/home.do), the mirDIP database (http://ophid.utoronto.ca/mirDIP/index.jsp#r), and the miRDB database (http://www.mirdb.org/). **b** Binding site between miR-503-5p and SGK1. **c** Expression of SGK1 in OA samples and normal samples in microarray dataset GSE46750. **d** Binding relationship between miR-503-5p and SGK1 confirmed by dual luciferase reporter gene assay. **e** RT-qPCR determination of SGK1 expression in chondrocytes after overexpressing/silencing miR-503-5p. **f** Western blot analysis of SGK1 expression in chondrocytes after overexpressing/silencing miR-503-5p. *p < 0.05. ^#^*p* < 0.05. Measurement data were expressed as mean ± standard deviation. Comparisons among multiple groups were conducted by one-way ANOVA with Tukey’s post hoc test. The experiment was repeated three times

The miRanda, mirDIP, and miRDB databases were used to predict the target genes of miR-503-5p, followed by intersection analysis with the upregulated genes in GSE46750. We obtained two genes, SGK1 and TMEM100 (Fig. 5a, b). Bioinformatics suggested upregulated SGK1 expression in OA sample (Fig. 5c). The results of luciferase assay verified that overexpression of miR-503 in hek293t inhibited the luciferase activity of SGK1 3′UTR-WT but had no effect on SKG1 3′UTR-MUT (Fig. 5d). Additionally, expression of SGK1 was reduced in chondrocytes from rats treated with overexpressed miR-503-5p but was elevated by silencing of miR-503-5p (Fig. 5e, f). All these results validated that miR-503 negatively regulated SGK1 expression.

### HDAC2 elevates SGK1 expression via reducing miR-503-5p expression to accelerate OA in rats

**Fig. 6:**
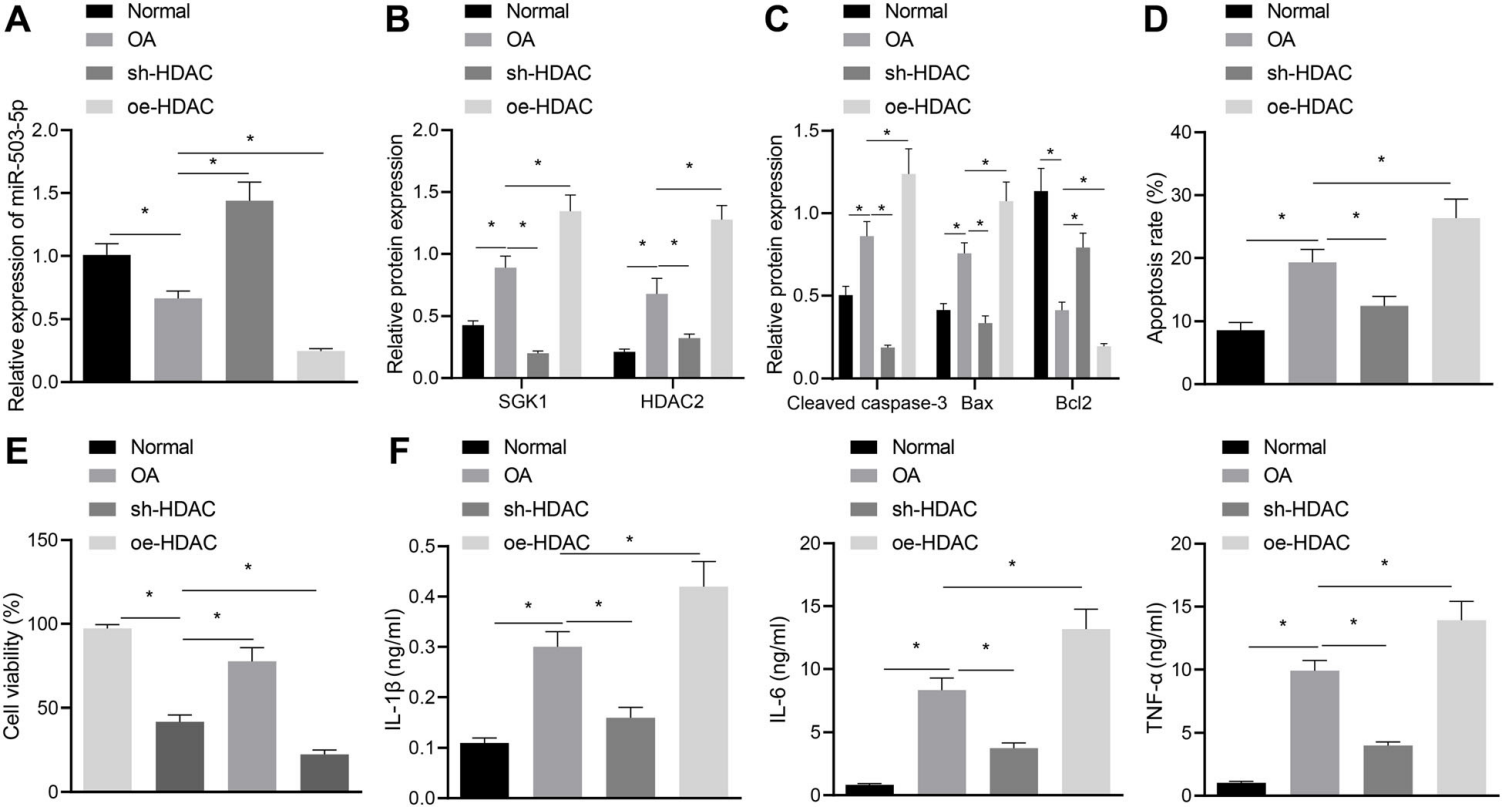
HDAC2 suppresses the proliferation of chondrocytes in OA rats through miR-503-5p/SGK1 axis. **a** Expression of miR-503-5p in primary chondrocytes measured by RT-qPCR assay. **b** Expression of SGK1 and HDAC2 in primary chondrocytes of HDAC2, normalized to GAPDH, determined by Western blot analysis. **c** The protein expression of apoptosis-related factors (Caspase-3, Bax, and Bcl2) in OA chondrocytes transfected with overexpressed or under-expressed HDAC2 measured by Western blot analysis. **d** Apoptosis in OA chondrocytes transfected with overexpressed or under-expressed HDAC2 detected by flow cytometry. **e** Proliferation of primary chondrocytes in OA rats treated with upregulated or downregulated HDAC2 assessed by MTT assay. **f** The expression of inflammatory cytokines in primary chondrocytes examined by ELISA. *p < 0.05 vs. normal chondrocytes. Measurement data were expressed as mean ± standard deviation. Comparisons among multiple groups were conducted by one-way ANOVA with Tukey’s post hoc test. The experiment was repeated three times

HDAC2 and SGK1 have been demonstrated to be highly expressed in OA [11, 15]. HDAC2 has been proved to downregulate miR-503-5p in this study. Next, we found that silencing HDAC2 inhibited the expression of miR-503-5p but enhanced SGK1 expression, while overexpression of HDAC2 led to the opposite results (Fig. 6a, b). The results of Western blot analysis (Fig. 6c), flow cytometry (Fig. 6d), MTT (Fig. 6f), and ELISA assay (Fig. 6g) further proved that upregulation of HDAC2 could downregulate miR-503-5p expression and upregulate SGK1 expression to inhibit proliferation and induce apoptosis of chondrocytes as well as promote secretion of inflammatory cytokines.

## Discussion

**Fig. 7:**
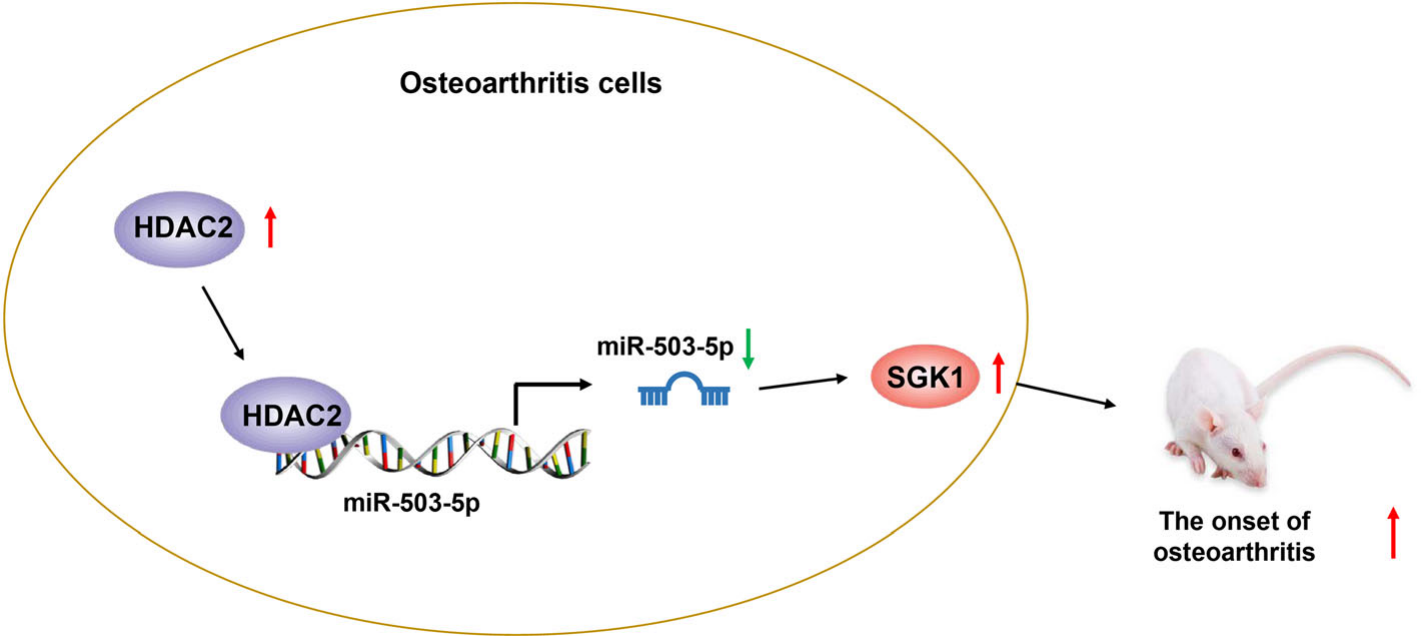
Schematic representation of functions of HDAC2, miR-503-5p, and SGK1 in OA. HDAC2 downregulates miR-503-5p expression to elevate SGK1 expression, thereby promoting OA in rats

It is known that chondrocyte apoptosis and inflammation is the most common pathological features of OA. Thus, targeting apoptosis and inflammatory pathways in chondrocytes may be a promising strategy for treating OA [17, 18]. More importantly, multiple lines of evidence have revealed the involvement of miRNAs in OA progression by regulating chondrocyte apoptosis and inflammation [6]. However, the specific mechanism of miR-503-5p in OA remains poorly understood. Thus, the effects of miR-503-5p on proliferation, apoptosis, and inflammation of chondrocytes were explored. The conclusion of this study demonstrated that HDAC2-suppressed miR-503-5p could reduce the expression of SGK1, thereby inhibiting the development of OA by facilitating proliferate and suppressing apoptosis and inflammation of chondrocytes in OA (Fig. 7).

miRNAs play an integral role in promoting osteogenesis as well as bone homeostasis, targeting which may be a potential treatment for osteoporosis or other bone diseases [19]. A finding obtained from a study has demonstrated low expression of miR-503 in patients with OA [20]. Interestingly, the first finding of the present study was that miR-503-5p was lowly expressed in tissues from patients from OA. Additionally, we also found that overexpression of miR-503-5p is able to promote the proliferation and inhibit the apoptosis of rat primary chondrocytes, as well as suppress inflammation, as evidence by reductions in expression of Caspase-3, Bax, and inflammatory cytokines and elevations in expression of Bcl2. Caspase-3, a member of cysteine proteases family, is responsible for most of the proteolysis during apoptosis [21]. Bax is a pro-apoptotic protein in the Bcl-2 family, which is upregulated in chondrocytes of patients with OA [22]. Bcl2 is an anti-apoptosis protein, whose alteration plays a key role in OA development [23, 24]. It has been documented that inhibition of Caspase-3 as well as Bax and stimulation of Bcl2 can suppress apoptosis of chondrocytes in OA [25, 26]. Besides, pro-inflammatory cytokines are upregulated in chondrocytes of OA [27]. The activation of pro-inflammatory cytokines reveals the pro-inflammatory process in the pathogenesis of OA [28, 29]. In line with our study, Li et al. also pointed out that miR-503 could suppress osteosarcoma cell invasion and metastasis [30].

Moreover, miR-503 targeting SGK1 was confirmed by luciferase test in the present study, and upregulation of miR-503 is capable of downregulating SGK1 expression. It is reported that miR-503-3p has tumor-suppressing properties in lung cancer by targeting p21 [31]. In addition, miR-503-5p has been proved to decrease the sensitivity of colorectal carcinoma to drug by inhibiting PUMA expression [32]. Similarly, our results revealed that miR-503-3p could lead to promoted proliferation and suppress apoptosis of rat primary chondrocytes, as well as inhibited inflammation by targeting and downregulating SGK1. High expression of SGK1 has been observed in chondrocytes of OA and suppression of SGK1 is able to restrain IL-1β-induced chondrocyte anabolic and catabolic imbalance in chondrocytes [12]. Furthermore, HDAC2 is able to mediate the suppression of cartilage-specific genes in human chondrocytes [33]. A corroborating study previously suggested that miR-503 is downregulated by HDAC2 through binding to the promoter of miR-503-5p and inhibiting H3K27ac expression [14]. Together with our experiment, we reported that HDAC2 downregulated miR-503-5p so as to upregulate SGK1 expression. Besides, overexpressed HDAC2 resulted in promoted proliferation and decreased apoptosis of chondrocytes, as well as enhanced inflammation through downregulation of miR-503-5p and elevation of SGK1 expression. Interestingly, there is a study indicating that HDAC2 is involved in the hypertrophic phenotype of cartilage [14]. More importantly, overexpressed HDAC2 has been proved in patients with OA and inhibition of HDAC2 is conductive to the development of cartilage [34].

## Conclusions

To briefly conclude, we demonstrated that the upregulation of HDAC2 promoted the apoptosis and inflammation of chondrocytes and inhibited proliferation in OA via the elevation of SGK1 by decreasing the expression of miR-503-5p. Therefore, silencing of HDAC2 or overexpressing of miR-503-5p may contribute to the treatment of OA.

## Data Availability

The datasets generated during the current study are available.

## Abbreviations

OA: Osteoarthritis
HDACs: Histone deacetylases
miR-503-5p: MicroRNA-503-5p
SGK1: Serum- and glucocorticoid-inducible kinase-1
RT-qPCR: Reverse transcription quantitative polymerase chain reaction
HE: Hematoxylin and eosin
